# Intrastromal autologous buffy coat as an adjuvant therapy for fungal corneal ulcer: a case report of two cases

**DOI:** 10.22336/rjo.2024.80

**Published:** 2024

**Authors:** Dharamveer Singh Choudhary, Kavita Ghanolia, Jeba Shaheen, Sidhya Choudhary, Anamika Chaudhary, Bhuvanesh Sukhlal Kalal

**Affiliations:** 1 Department of Ophthalmology, Swai Man Singh Medical College and Hospitals, Jaipur, Rajasthan, India; 2 Department of Microbiology, Swai Man Singh Medical College and Hospitals, Jaipur, Rajasthan, India; 3 Department of Pathology, Swai Man Singh Medical College and Hospitals, Jaipur, Rajasthan, India; 4 Department of Pharmacology and Nutritional Sciences, College of Medicine, University of Kentucky, Lexington, Kentucky, USA

**Keywords:** buffy coat, fungal ulcer, corneal ulcer, intrastromal

## Abstract

**Purpose:**

To report the hastened recovery of two nonresponding fungal corneal ulcer cases after using intrastromal injections of autologous buffy coat (a component of blood rich in leucocytes) as an adjuvant to topical antifungal therapy.

**Methods:**

Microbiologically proven non-responding cases (>2 weeks of treatment) of fungal corneal ulcers who visited the cornea specialty clinic of our hospital were explained the disease and adjuvant intrastromal autologous buffy coat treatment, which would be repeated if effective. Then, well-informed and written consent was obtained from the patients for the treatment. The study adhered to the tenets of the Declaration of Helsinki. An in vitro experiment was also carried out to prove the hypothesis by growing fungi on a donor button and injecting it with the buffy coat of a healthy volunteer.

**Results:**

The study showed immediate positive outcomes, with patients showing faster lesion resolution with decreasing lesion size, resolving hypopyon, and complete clinical recovery in both cases in less than seven days.

**Discussion:**

Intrastromal autologous buffy coat shows promise as an adjunct therapy for difficult fungal corneal ulcers, enhancing local immunity and healing. This approach may reduce treatment time and risks, though further studies are needed to confirm these findings.

**Conclusion:**

Intrastromal buffy coat for recalcitrant fungal ulcers is a promising treatment modality as it provides both local immunity and growth and repair stimulus to the diseased cornea. However, a long-term randomized control trial is needed to evaluate the results further.

## Introduction

Fungal corneal ulcers are one of the common corneal ulcers and cause long-term morbidity, especially in rural populations where agriculture is the main occupation and medical facilities are scarce, especially in the developing world. Managing fungal corneal ulcers has proven challenging due to the cornea’s poor vascular supply, dry eyes, poor availability, penetration, limited spectrum of activity, and surface toxicity of drugs [1]. The ophthalmologist also has surgical treatment methods in his arsenal. However, even surgical treatment is not definitive and cannot fully replace topical management due to high costs, post-surgical complications, recurrence, and limited availability of corneas [2]. The natural course of healing these corneal ulcers is slow corneal perforation followed by iris plugging, which serves the affected part with sufficient blood vessels and natural defense, eradicating fungus and healing by forming an adherent leucoma [3]. Surgical grafting and conjunctival hooding have also been done for a very long time to provide vascular supply to the lesion [4]. This prompted us to plan a supply of fresh macrophage-rich components of autologous blood directly on the lesion by intrastromal route (about two units of insulin syringe) as is done with voriconazole and amphotericin B. Hence, we studied autologous buffy coats in patients nonresponsive to topical antifungal therapy for their treatment.

## Case 1

**Fig. 1:**
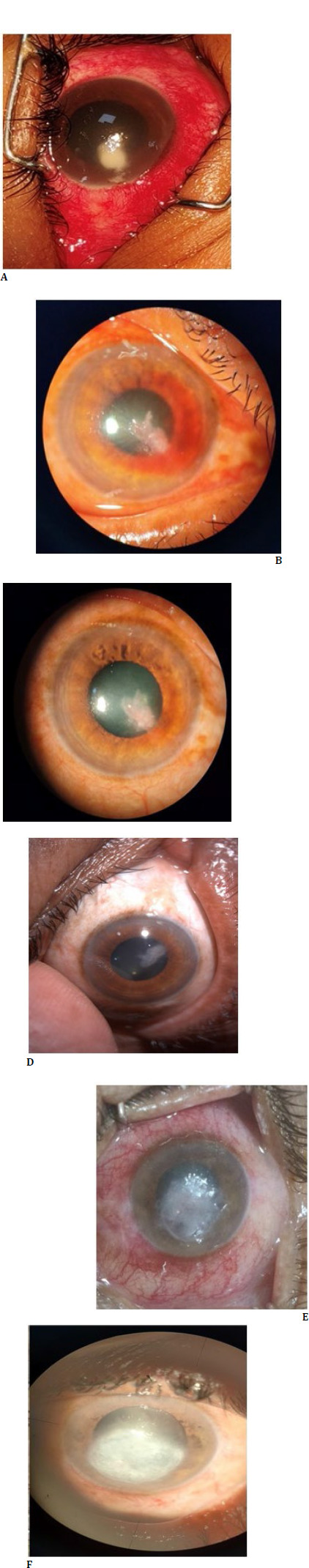
Serial of photographs showing injection healing of case 1 (pre **A** and post **B, C, D**) and case 2 (pre **E** and post **F**).

A 32-year-old male presented to the outpatient department (OPD) with a history of fall of vegetative matter in his left eye while working in the field approximately 20 days back. The patient complained of dryness, mild pain, watering, and redness in the right eye (**Fig. 1 A**). The vision was 20/200 in the left eye. The patient had a dry-looking lesion with ulceration and infiltrates. The lesion on the slit lamp was located in the anterior half of the stroma, and the size was 4×2 mm. The patient on scrapping was found to be infected with Aspergillus species. The patient was non-responsive to topical medication. Then, he was started on adjunctive intrastromal treatment once daily. There was a considerable improvement after the first injection on day one, both symptomatic and on slit lamp examination (**Fig. 1 B, C**). After the three successive intrastromal injections, there was a complete recovery, and the lesion healed with a small scar (**Fig. 1 D**).

## Case 2

A 55-year-old male presented with a grossly round lesion approximately 4×4 mm in size located in the pupillary area and limited to half of the anterior stroma (**Fig. 1 E**) after 20 days. On examination, vision in the affected eye (left) was hand movement close to face (HMCF), PL+, PR accurate. His corneal scrapping was fusarium positive, barely responding to topical antifungals (both natamycin 5% and Voriconazole). He was then explained about adjuvant autologous intrastromal buffy coat therapy or intrastromal voriconazole therapy to hasten recovery. He then opted for the formal one. There was a considerable improvement both in the lesion and symptomatically after the first injection, and the ulcer healed entirely with scarring, as shown in **Fig. 1 F**, after three consecutive injections of buffy coat in three days.

## Results

In both cases, intrastromal injections of autologous buffy coat demonstrated rapid and significant clinical improvements. The first case, a 32-year-old male with a fungal corneal ulcer unresponsive to two weeks of topical antifungal treatment, showed marked improvement in lesion size and symptoms within one day of the initial injection, ultimately achieving full recovery with minimal scarring after three consecutive injections. Similarly, in the second case, a 55-year-old male with a Fusarium-positive ulcer displayed a notable reduction in lesion size and symptom relief within one day following the initial buffy coat injection. Complete healing was achieved after three injections over three days, resulting in scar formation without further complications. These findings suggest that intrastromal buffy coat injections can expedite recovery in recalcitrant fungal corneal ulcers, potentially providing enhanced local immunity and reparative effects at the site of infection.

## Discussion

The treatment of fungal corneal ulcers has always been a challenge. Many times, the ulcer becomes refractory to the treatment given, or it can take months for the disease to show signs of resolution, even when the right medical therapy is followed meticulously. All this can be attributed to the fact that the drugs administered have poor penetration, limited spectrum of activity, and surface toxicity [1].

The other option after medical therapy fails is surgery [5]. However, drug therapy is more economical and also does not have the risk of graft rejection [2,5], thus making this more feasible for the patient and saving them from physical pain and the financial burden that comes with surgery.

We dealt with the problem of non-resolving corneal fungal infection by directly providing local immunity and healing stimulus at the ulcer site through an intrastromal buffy coat. The buffy coat is the part of centrifuged blood that contains the maximum concentration of leucocytes and platelets [6-8]. So, the intervention essentially involves providing pre-formed immunity to the diseased area through leucocytes and growth factors from the platelet part that help in healing.

Other interventions in intrastromal voriconazole [9] have shown promise but do not simultaneously provide immunity and healing to treat the disease. There has also been a single study involving intrastromal injected autologous serum [10], but using a buffy coat for treatment is a first of its kind. Even though both autologous serum and buffy coat have similarities, their comparability cannot be commented on as there is no study for that. Moreover, positive results were obtained for both [8].

**Fig. 2:**
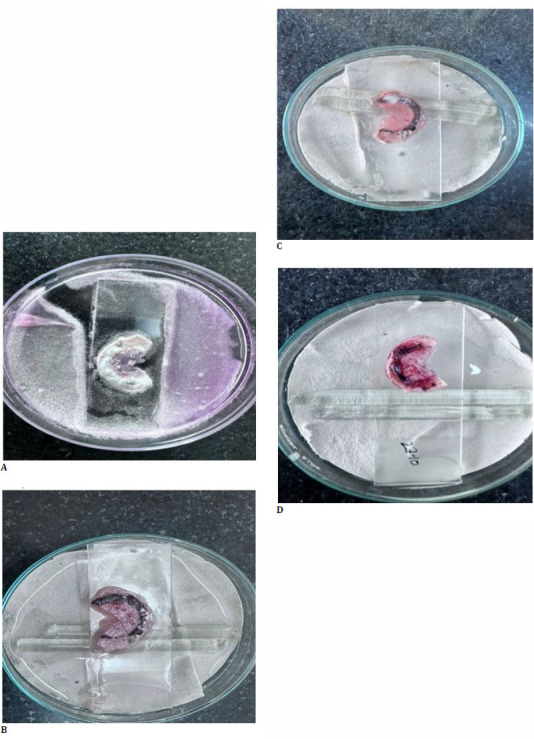
*In vitro* culture of a corneal button with Aspergillus species and intrastromal buffy coat treatment. Corneal button infection with aspergillus species at day 2 (**A**), treatment of buffy coat at day 1 (**B**), recovery at day 2 (**C**), and complete healing at day 5 (**D**).

To further prove our hypothesis, we experimented in vitro. We inoculated the hemi-cut donor corneal button with aspergillus species and waited two days (**Fig. 2 A**). After complete growth, we inoculated this button with a buffy coat from a healthy volunteer. **Fig. 2 B** depicts changes brought by the intrastromal buffy coat after one day. One can see near-complete tissue recovery and fungal load subsiding heavily. On day 5 (i.e., two days after the results of the intrastromal buffy coat), some fungal load reappeared due to the germination of surviving spores (**Fig. 2 C**). We further repeated the same injection of intrastromal buffy coat, and **Fig. 2 D** shows the outcome of complete recovery from fungus.

The study’s drawbacks were its size and the long-term follow-up for any adverse effects related to the treatment, for which a larger sample size and longer duration are needed.

## Conclusion

Intrastromal buffy coat for recalcitrant fungal ulcers is a promising treatment modality as it provides both local immunity and growth and repair stimuli to the diseased cornea. However, long-term randomized controlled trials are required to evaluate these results further.
